# Isoform-Specific Reduction of the Basic Helix-Loop-Helix Transcription Factor TCF4 Levels in Huntington’s Disease

**DOI:** 10.1523/ENEURO.0197-21.2021

**Published:** 2021-10-13

**Authors:** Kaja Nurm, Mari Sepp, Carla Castany-Pladevall, Jordi Creus-Muncunill, Jürgen Tuvikene, Alex Sirp, Hanna Vihma, Derek J. Blake, Esther Perez-Navarro, Tõnis Timmusk

**Affiliations:** 1 Department of Chemistry and Biotechnology, Tallinn University of Technology, Tallinn 12618, Estonia; 2 Protobios LLC, Tallinn 12618, Estonia; ^3^Departament de Biomedicina, Facultat de Medicina i Ciències de la Salut, Institut de Neurociències, Universitat de Barcelona, Barcelona, Catalonia 08036, Spain; 4 Institut d’Investigacions Biomèdiques August Pi i Sunyer (IDIBAPS), Barcelona, Catalonia 08036, Spain; 5 Centro de Investigación Biomédica en Red sobre Enfermedades Neurodegenerativas (CIBERNED), Madrid, 28031, Spain; 6 Division of Psychological Medicine and Clinical Neurosciences, MRC Centre for Neuropsychiatric Genetics and Genomics, Cardiff University, Cardiff CF24 4HQ, United Kingdom

**Keywords:** basic helix-loop-helix transcription factor, Huntington’s disease, neurodegenerative disease, TCF4, transcriptional regulation

## Abstract

Huntington’s disease (HD) is an inherited neurodegenerative disorder with onset of characteristic motor symptoms at midlife, preceded by subtle cognitive and behavioral disturbances. Transcriptional dysregulation emerges early in the disease course and is considered central to HD pathogenesis. Using wild-type (wt) and HD knock-in mouse striatal cell lines we observed a HD genotype-dependent reduction in the protein levels of transcription factor 4 (TCF4), a member of the basic helix-loop-helix (bHLH) family with critical roles in brain development and function. We characterized mouse *Tcf4* gene structure and expression of alternative mRNAs and protein isoforms in cell-based models of HD, and in four different brain regions of male transgenic HD mice (R6/1) from young to mature adulthood. The largest decrease in the levels of TCF4 at mRNA and specific protein isoforms were detected in the R6/1 mouse hippocampus. Translating this finding to human disease, we found reduced expression of long TCF4 isoforms in the postmortem hippocampal CA1 area and in the cerebral cortex of HD patients. Additionally, TCF4 protein isoforms showed differential synergism with the proneural transcription factor ASCL1 in activating reporter gene transcription in hippocampal and cortical cultured neurons. Induction of neuronal activity increased these synergistic effects in hippocampal but not in cortical neurons, suggesting brain region-dependent differences in TCF4 functions. Collectively, this study demonstrates isoform-specific changes in TCF4 expression in HD that could contribute to the progressive impairment of transcriptional regulation and neuronal function in this disease.

## Significance Statement

Historically, Huntington’s disease (HD) has been considered a neurodegenerative disease. However, research of the last decade has revealed disrupted neurogenesis and cognitive dysfunction preceding pathologic neuronal cell death, suggesting that HD is also a neurodevelopmental disease. One of the major molecular mechanisms of HD is dysregulation of transcription. Studying transcription factors with functions in neurogenesis and neural plasticity is of interest for their potential participation in the cognitive impairment in HD etiology. Here, we show reduced expression of the transcription factor TCF4, previously linked with neurodevelopmental and neuropsychiatric diseases, in hippocampus and cerebral cortex of R6/1 mouse and HD patients. Our results shed light on the potential neurodevelopmental aspect of HD and could be applicable for developing alleviating therapies for HD.

## Introduction

Huntington’s disease (HD; OMIM #143100) is a fatal inherited neurodegenerative disorder caused by autosomal dominant mutation in the huntingtin (*HTT*) gene (The Huntington’s Disease Collaborative Research Group, 1993). The mutation is a trinucleotide repeat (CAG)_n_ expansion in *HTT* exon 1, leading to translation of abnormal HTT protein with expanded polyglutamine tract in its N terminus. To study the disease mechanisms, different HD models have been developed, including chemically induced models using mitochondrial toxin 3-nitropropionic acid (3-NP) and genetic models in various cell-based systems and animals (for review, see Pouladi et al., 2013).

Clinically, HD is characterized by chorea and impairment of voluntary movements caused by striatal and cortical neurodegeneration that are preceded by cognitive and psychiatric disturbances (Stout et al., 2011). However, based on the critical functions of HTT in the developing nervous system a developmental basis for HD has been also proposed (Zuccato and Cattaneo, 2014; Bates et al., 2015). In fact, human and mouse embryos carrying mutant *HTT* show clear abnormalities in the developing cortex, including defects in neural progenitor differentiation and cell cycle progression (Barnat et al., 2020). Moreover, carriers of mutant *HTT* gene have changes in the striatum (van der Plas et al., 2019) and cerebellum (Tereshchenko et al., 2020) at presymptomatic stages.

In healthy adults, cognitive functions such as learning and memory rely on neuronal plasticity and neurogenesis in the hippocampus and cerebral cortex (McClelland et al., 1995; Chambers et al., 2004; Aimone et al., 2009; Gonçalves et al., 2016; Mansvelder et al., 2019). While atrophy and neurodegeneration in striatum and cerebral cortex is a neuropathological hallmark of HD, the hippocampus remains relatively unaffected in HD patients (Ramirez‐Garcia et al., 2020), although misregulated transcriptional pathway of synaptic vesicles has been observed (Neueder and Bates, 2014). On the other hand, reduced hippocampal adult neurogenesis in subgranular zone (SGZ) of the dentate gyrus (DG) has been reported in R6/2, R6/1, and YAC128 HD mouse models (Lazic et al., 2004; Gil et al., 2005; Simpson et al., 2011) and Q175FDN knock-in HD mice show deficits in hippocampal synaptic plasticity (Quirion and Parsons, 2019).

Transcriptional dysregulation is a well-characterized molecular mechanism in HD pathophysiology (for review, see Sugars and Rubinsztein, 2003; Cha, 2007; Valor, 2015). Mutant HTT has been shown to bind and disrupt the functions of general transcription factors and transcriptional regulators in HD (Dunah et al., 2002; Luthi-Carter et al., 2002; Bae et al., 2005; Zhai et al., 2005) and has impaired capacity to interact with RE1-silencing transcription factor/neuron-restrictive silencer factor (REST/NRSF), which alters the expression of many neuronal genes including brain-derived neurotrophic factor (BDNF; Zuccato et al., 2003, 2007). Furthermore, the global transcriptional dysregulation in HD has been refined in genomics, proteomics and network analysis studies (Langfelder et al., 2016; Hensman Moss et al., 2017; Ament et al., 2018).

Transcription factor 4 (TCF4) belongs to a large bHLH transcription factor family and is a dimerization partner to other bHLH proteins for binding to E-box sequences of target genes (for review, see Massari and Murre, 2000). Importantly, TCF4 should not be confused with key factor of Wnt signaling pathway TCF7L2, unofficially called TCF-4. *TCF4* is widely but not equally expressed in human brain and the use of numerous alternative 5′ exons leads to generation of many different TCF4 protein isoforms (Sepp et al., 2011). TCF4 regulates many genes implicated in neurodevelopment, ion channel functions and signal transduction (Forrest et al., 2018). Furthermore, TCF4 regulates synaptic plasticity and memory, and overexpression of TCF4 results in abnormal distribution of layer 2/3 pyramidal neurons in prefrontal cortex and alters their intrinsic excitability (Kennedy et al., 2016; Page et al., 2018; Thaxton et al., 2018). A recent study by Tamberg and colleagues (Tamberg et al., 2020) showed that reduction of Daughterless (*Drosophila melanogaster* homolog of TCF4) in larvae central nervous system impairs appetitive associative learning and downregulates synaptic protein encoding genes, therefore linking Daughterless, and possibly TCF4, to memory formation (Tamberg et al., 2020).

Our immunocytochemical screen of different transcription factors in a cellular model of HD revealed differential expression and/or localization of TCF4. Therefore, based on the developmental hypothesis as well as disturbed synaptic plasticity and adult neurogenesis in HD, we decided to thoroughly investigate the expression of neurodevelopmentally important TCF4 in cellular and mouse models of HD and in HD patients. We show that TCF4 levels are reduced in HD brain in an isoform-dependent and brain region-dependent manner. We further reveal differences in the ability of different TCF4 isoforms to cooperate with Class II bHLH transcription factor ASCL1 in neurons. These results implicate reduced and/or imbalanced TCF4 function as a possible factor in HD etiology.

## Materials and Methods

### Cell culture

The striatal progenitor Hdh^7/7^, Hdh^7/109^, and Hdh^109/109^ cell lines have been described previously (Zuccato et al., 2003). Briefly, Hdh^7/7^ cells are derived from wild-type (wt) mice carrying two copies of the endogenous *Htt* alleles with 7 CAG repeats; Hdh^7/109^ and Hdh^109/109^ are derived from heterozygous and homozygous *knock-in* mice with one or both *Htt* alleles containing 109 CAG repeats, respectively. Hdh cells were propagated in DMEM (Invitrogen) supplemented with 10% fetal bovine serum (PAA Laboratories), 100 U/ml penicillin and 0.1 mg/ml streptomycin (PAA Laboratories) at 33°C in 5% CO_2_.

Separate rat cortical and hippocampal neuronal cultures were prepared from embryonic day (E) 21 Sprague Dawley rat embryos as described previously (Esvald et al., 2020). Where indicated, neurons were treated with 0.5 mm 3-NP (Sigma-Aldrich) for 0–16 h at 6–8 days *in vitro* (DIV).

### Mice

Male R6/1 transgenic mouse (B6CBA background) expressing the N-terminal exon 1 fragment of mutant *HTT* with 115 CAG repeats (obtained originally from The Jackson Laboratory) and their wt littermate controls were used for this study. Genotyping was performed by PCR from tail biopsy samples using primers designed for the expansion of the exon 1 of the mutant *HTT* as previously described (Mangiarini et al., 1996). All mice were housed together in numerical birth order in groups of mixed genotypes with access to food and water *ad libitum* in a colony room kept at 19–22°C and 40–60% humidity, under a 12/12 h light/dark cycle. Mice were exsanguinated at 8, 12, 20, and 30 weeks of age and brain was quickly removed for cortex, hippocampi and striata dissection. All procedures were conducted in accordance with the National Institutes of Health *Guide for the Care and Use of Laboratory Animals*, and approved by the local animal care committee of the Universitat de Barcelona, following European (2010/63/UE) and Spanish (RD53/2013) regulations for the care and use of laboratory animals.

### Postmortem human brain tissue

Frozen samples of hippocampus and cerebral cortex from HD patients and control individuals were obtained from the Neurologic Tissue Bank of the Biobank-Hospital Clínic-Institut d’Investigacions Biomèdiques August Pi i Sunyer (IDIBAPS) following the guidelines and approval of the local ethics committee (Hospital Clínic of Barcelona’s Clinical Research Ethics Committee). Informed consent was obtained from all subjects, and experiments were performed following the guidelines and approval of the local ethics committee. Details on the sex, age, CAG repeat length, Vonsattel grade, and postmortem delay are found in Table 1.

**Table 1:** Hippocampus CA1 and cerebral cortex tissue samples of HD patients

ID	Region	Pathologic diagnosis	Gender	Age (years)	CAG repeats	PMD (h:min)*
CS-497	CA1	Control	M	82		02:30
CS-1694	CA1	Control	M	58		05:00
CS-1858	CA1	Control	F	83		07:30
CS-1870	CA1	Control	F	97		07:20
CS-1888	CA1	Control	F	93		05:30
CS-1937	CA1	Control	F	83		07:33
CS-1949	CA1	Control	M	86		07:25
CS-1193	CA1	HD, Vonsattel grades 3–4	M	55	-	07:00
CS-1294	CA1	HD, Vonsattel grade 3	M	53	45 ± 2	07:00
CS-1334	CA1	HD, Vonsattel grade 1	M	73	40 ± 2	07:00
CS-1438	CA1	HD, Vonsattel grade 3	M	85	40	05:30
CS-1630	CA1	HD, Vonsattel grade 2	M	76	41	06:00
CS-1638	CA1	HD, Vonsattel grade 2	M	72	-	13:10
CS-1844	CA1	HD, Vonsattel grade 2	F	69	43	15:30
CS-1874	CA1	HD, Vonsattel grade 3	M	56	43	04:30
CS-1875	CA1	HD, Vonsattel grades 2–3	M	84	39	08:00
CS-1933	CA1	HD, Vonsattel grade 2	F	86	40	12:20
810	Cortex	Control	F	81		23:30
1679	Cortex	Control	F	90		12:20
1697	Cortex	Control	M	78		06:00
1870	Cortex	Control	F	97		07:20
1937	Cortex	Control	F	83		07:33
1949	Cortex	Control	M	86		07:25
1980	Cortex	HD, Vonsattel grade 3	F	69	-	12:30
1875	Cortex	HD, Vonsattel grades 2–3	M	84	39	08:00
1844	Cortex	HD, Vonsattel grade 2	F	69	43	15:30
1638	Cortex	HD, Vonsattel grade 2	M	72	-	13:10
1630	Cortex	HD, Vonsattel grade 2	M	76	38	06:00
909	Cortex	HD, Vonsattel grade 4	M	60	43	13:30

Details of human postmortem samples used for Western blotting to analyze protein levels of TCF4. All samples received from the Neurologic Tissue Bank-Biobanc located at the University of Barcelona School of Medicine. *PMD, postmortem delay.

### Immunocytochemistry

Hdh cells grown on poly-L-lysine-coated coverslips were fixed and treated as previously described (Kannike et al., 2014). Rabbit polyclonal anti-TCF4 (#HLH201, CeMines) and other polyclonal antibodies (Extended Data Fig. 1-1, CeMines) were diluted 1:200 and Alexa Fluor 488-conjugated goat anti-rabbit IgG antibodies (Invitrogen) were diluted 1:2000 in 0.2% BSA and 0.1% Tween 20 in PBS. DNA was counterstained with DAPI included in the mounting medium (ProLong Gold Antifade mountant with DAPI; Thermo Fisher). The specificity of the nuclear signal of the used TCF4 antibody has been validated previously (Sepp et al., 2017).

### Western blotting

Hdh cells and rat cultured cortical neurons were lysed in RIPA buffer (50 mm Tris-HCl, pH 8, 150 mm NaCl, 1% NP-40, 0.5% sodium deoxycholate, 0.1% SDS, 1 mm dithiothreitol and protease inhibitors cocktail Complete mini; Roche). Cell lysates were sonicated for 15 s with 30% amplitude on Sonics VibraCell and centrifuged at 16,100 × *g* for 15 min at 4°C. Nuclear and cytosolic fractions of Hdh cells were prepared with NE-PER Nuclear and Cytoplasmic Extraction kit (Thermo Scientific) according to the manufacturer’s instructions. Protein concentrations in lysates were measured with BCA Protein Assay kit (Pierce).

Protein extraction from mouse brain and human postmortem samples from the cerebral cortex and CA1 region of the hippocampus was performed using a lysis buffer containing 50 mm TRIS (pH 7.4), 10% glycerol, 1% Triton X-100, 150 mm NaCl, 5 μm ZnCl_2_, 10 mm EGTA and protease inhibitors [2 mm phenylmethylsulfonyl fluoride (PMSF), 10 μg/μl aprotinin, and 1 μg/μl leupeptin] and phosphatase inhibitors (2 mm Na_3_VO_4_ and 100 mm NaF). Samples were homogenized and supernatants were collected after centrifugation at 15,000 × *g* for 15 min at 4°C. Protein concentration was measured using Dc protein assay kit (Bio-Rad Laboratories).

TCF4 isoforms A^–^, B^–^, C^–^ and D^–^ were *in vitro* translated using TnT Quick Coupled Transcription/Translation System (Promega) according to the manufacturer’s instructions. The used DNA constructs have been described previously (Sepp et al., 2011). The frontal cortex lysates from E18 *Tcf4* knock-out (KO) and wt mice were a kind gift from Brady Maher and have been described in more detail previously (Li et al., 2019).

Equal amounts of protein were separated in 8% gel in SDS-PAGE followed by wet transfer to Hybond-C nitrocellulose membrane (GE Healthcare) or to PVDF membrane (Merck Millipore). Membranes were blocked 1 h at room temperature (RT) in PBS containing 5% skimmed milk and 0.1% Tween 20, incubated overnight at 4°C with primary antibodies and 1 h at RT with secondary antibodies in 0.1% Tween 20 (and 5% BSA for the mouse and postmortem human brain samples) and 2% skimmed milk in PBS. Antibodies were diluted as follows: 1:200 rabbit polyclonal anti-TCF4 (TCF4_02; 200 ng/ml; Forrest et al., 2018), 1:1000 mouse monoclonal anti-TCF4 (ITF-2 C-8; #sc-393407; 200 μg/ml; Santa Cruz), rabbit polyclonal anti-HDAC2 1:1000 (#sc-7899, Santa Cruz), mouse monoclonal anti-β tubulin to final concentration 30 ng/ml (E-7, DSHB), anti-α tubulin (T9026, Sigma-Aldrich) and HRP-conjugated goat anti mouse or rabbit IgG (H+L) 1:2500 (#32430 and #32460, respectively; Thermo Scientific). Chemoluminescence signal was detected using SuperSignal West Femto Chemoluminescent Substrate (Thermo Scientific) and ImageQuant 400 imaging system (GE Healthcare). Additionally, Coomassie staining was used to quantify loading in five independent Western blotting experiments with Hdh total lysates. Briefly, the membrane was stained with Coomassie solution (0.1% Coomassie Brilliant Blue R-250 dye, 25% ethanol, 7% acetic acid), followed by two washes with destaining solution (30% ethanol, 10% acetic acid) and rinsing with tap water. Densitometric quantification was done using ImageQuant T4 v2005 software (GE Healthcare) or Gel-Pro Analyzer version 4 (Media Cybernetics).

### TCF4 transcripts data mining and visualization

Mouse *Tcf4* gene structure and mRNAs were identified by analyzing genomic, mRNA and expressed sequence tag (EST) databases available at https://genome.ucsc.edu/ and https://www.ncbi.nlm.nih.gov/nuccore. Accession numbers of representative mouse mRNA or EST sequences for alternative *Tcf4* transcripts are shown in Table 2, based on Mouse Dec. 2011 GRCm38/mm10 genome assembly. Human transcript data adapted from Sepp et al. (2011) is included for reference (Human Dec. 2013, GRCh38/hg38 assembly).

**Table 2:** Accession numbers of representative mRNA or EST sequences for alternative *Tcf4* transcripts and protein isoforms they encode in mouse and human

Alternative TCF4 transcripts and isoforms			
Transcript	Protein isoform	Mouse	Human
3b	B	AK133885	AK315074
3bΔ3	C	AK051958	AK299169
3c	B	AK081012	DB106801
3cΔ3	E	ENSMUST00000202354.3	FR748216
3cΔ8-9	BΔ	ENSMUST00000202772.3	FR748212
3d	B	CJ182557	M74719
4c	C	CJ115804	DC358747
7a-II	D	BY247629	AK300612
7b-I	D/G	XM_017317861	AK095041
7b-II	D	CD350230	DC350124
8a	D	BY286412	AK316165
8b*/8b-I	D	CB178848	FR748208
8b-II	D	BY252182	AK300636
8c-II	D	BB663894	CA393351
8e/8d	D	BY259217	BP230382
10a	A	U16321	AK300038
10b	I	BU058820	BP241032
10c	H	BY333068	DA664480

Reference for transcripts of human *TCF4* were obtained from Sepp et al. (2011). Data are according to Mouse Dec. 2011 (GRCm38/mm10) Assembly and Human Dec. 2013, GRCh38/hg38 assembly. *TSS differs between human and mouse. The respective human transcript is shown after slash.

### RNA extraction and cDNA synthesis

Total RNA from Hdh cells and cultured cortical neurons was extracted using RNeasy Micro kit (QIAGEN) following the manufacturer’s instructions. R6/1 mouse brain tissue was homogenized in QIAZol reagent (QIAGEN) and total RNA was extracted with RNeasy Lipid Tissue Mini kit (QIAGEN) according to the manufacturer’s protocol including on-column DNase I treatment. First-strand cDNAs were synthesized from 1 to 5 μg of total RNA with Superscript III First-Strand synthesis system (Invitrogen) together with oligo(dT)_20_ or a combination of oligo(dT)_20_ and random hexamer primers (Microsynth) in case of cultured cells and R6/1 brain RNA, respectively.

### Quantitative PCR (qPCR)

qPCRs were performed in triplicates using LightCycler 480 SYBR Green I Master (Roche) with cDNA from Hdh cells and 3-NP-treated cultured cortical neurons and 5× HOT FIREPol EvaGreen qPCR Mix Plus (Solis Biodyne) with R6/1 mouse brain cDNAs on LightCycler 480 II Real Time PCR System (Roche). Levels of *Sdha* or *Hprt1* (for HD cell model experiments: Hdh and 3-NP-treated neurons, respectively) or geometric mean of *Hprt1*, *Gapdh*, and *Tbp* (for R6/1 mouse experiments) mRNA levels were used to normalize qPCR data. Primer pairs used in qPCR are shown in Table 3.

**Table 3:** List of oligonucleotides used in this study

Gene (transcript)	Forward primer sequence	Reverse primer sequence
*Tcf4 total*	TACGCTCCTTCAGCCAGCAC	TGGATGCAGGCTACAGTAGCTG
*Tcf4-B/C*	AGAAGACAGAAGTAGCTCAGGGTC	GTTTGGTGGGCGAAAGGGTTCC
*Tcf4-A*	caccATGTACTGCGCATACACCATC	TGGATGCAGGCTACAGTAGCTG
*Tcf4-B*	caccATGCATCACCAACAGCGAATGG	GGACCCTGAGCTACTTCTGTCTTC
*Tcf4-D (8c-II)*	CAGCTGAAATGATTCCCCACTGTG	TGGATGCAGGCTACAGTAGCTG
*Tcf4-D (7b-I)*	GTCTTGCTTGCATACATTGCCAG	GTTTGGTGGGCGAAAGGGTTCC
*Tcf4-I*	GAGAAAGCCCAAGTTAGGCTGAG	TGGATGCAGGCTACAGTAGCTG
*Bdnf*	GGCCCAACGAAGAAAACCAT	AGCATCACCCGGGAAGTGT
*Ascl1*	AACTCTATGGCGGGTTCTCCGGT	CTGCCATCCTGCTTCCAAAGT
*Neurod1*	ACACGAGGCAGACAAGAAgG	TCTTGGGCTTTTGATCaTCC
*Hprt1*	CAGTCCCAGCGTCGTGATTA	AGCAAGTCTTTCAGTCCTGTC
*Sdha*	AACACTGGAGGAAGCACAC	GGAACGGATAGCAGGAGGT
*Gapdh*	TGCACCACCAACTGCTTAGC	GGCATGGACTGTGGTCATGAG
*Tbp*	TGCACAGGAGCCAAGAGTGAA	CACATCACAGCTCCCCACCA

### Plasmid constructs

Expression constructs pcDNA.3.1/EF1a/TCF4-B^–^ and pcDNA.3.1/EF1a/TCF4-A^–^ have been characterized previously (Sepp et al., 2017). The constructs for TCF4-C^–^, TCF4-D^–^, and TCF4-I^–^ were created similarly from the respective pcDNA3.1 constructs as described (Sepp et al., 2011) by replacing the cytomegalovirus (*CMV*) promoter with elongation factor 1 (*EF1α*) promoter from pGL4.83[hRlucP/EF1α/Puro]. pcDNA3.1/PGK/ASCL1 was cloned from pcDNA3.1/SRα/ASCL1 (Sepp et al., 2017) replacing *SRα* promoter with *PGK* promoter from pGL4.83[hRlucP/PGK1/Puro]. Luciferase reporter system constructs pGL4.29[luc2P/12μE5/TK/Hygro] with 12 E-boxes in front of thymidine kinase promoter, and pGL4.83[hRlucP/PGK1/Puro] with phosphoglycerate kinase 1 promoter have been characterized previously (Sepp et al., 2011, 2017).

### Luciferase reporter assay

Neurons plated on 48-well plates were transfected at 6 DIV using Lipofectamine 2000 (Invitrogen) with a reagent to DNA ratio 3:1. For luciferase reporter assays, 0.06 μg of TCF4 isoforms B^–^, C^–^, D^–^, A^–^, and I^–^, and 0.06 μg of ASCL1 encoding constructs were used, 0.06 μg of firefly luciferase construct pGL4.29[luc2P/12μE5/TK/Hygro], and 0.02 μg of *Renilla* luciferase pGL4.83[hRlucP/PGK1/Puro] were used. At 7 DIV, the neuronal cultures were left untreated or treated with 25 mm KCl for 8 h. Cells were lysed in Passive Lysis buffer (Promega) and Dual-Glo Luciferase assay (Promega) was used for measuring luciferase signals. Luciferase assay was performed in technical duplicates and in total three independent experiments were conducted both on cortical and hippocampal primary neurons. Normalized luciferase data were used to calculated co-operation indices between TCF4 and ASCL1 as described (Chang et al., 1996). Briefly, co-operation index shows how many times the transactivation fold is increased in neurons co-expressing different TCF4 isoforms and ASCL1 compared with the sum of transactivation folds from neurons expressing both proteins separately.

### Statistical analysis

For quantification of Western blotting data TCF4 signals were normalized to the signals of β tubulin (Hdh whole and cytoplasmic lysates; Fig. 2*C*,*E*), HDAC2 (nuclear lysates; Fig. 2*E*), Coomassie staining (Hdh whole lysates; Fig. 2*C*), or α tubulin (lysates from human CA1 region of the hippocampus and cerebral cortex; Fig. 4*F*,*H*). The data were log-transformed to ensure normal distribution, autoscaled (where indicated in the figure legend), mean and SEM were calculated, and two-tailed *t* tests were used for statistical analysis (Extended Data Figs. 2-1, 4-1). The data were back-transformed into the linear scale for graphical representation, error bars represent upper and lower limits of the back-transformed mean ± SEM. For analysis of TCF4 protein levels in R6/1 mouse brain time series, the TCF4 signals were normalized to the signals of α tubulin, data were log-transformed, mean and mean ± SEM were calculated for each group, and the average expression level of the respective transcript in each age group was set as 0 (1 in linear scale). Generalized linear model with Gaussian distribution using the formula genotype + age:genotype was used, followed by Wald χ^2^ test (Type III test, performed with Car package in R; Fox and Weisberg, 2019) to determine *p* values of the coefficients. For graphical representation, data were back-transformed to the linear scale, with error bars showing back-transformed mean ± SEM. qPCR data generated from Hdh cell line and 3-NP-treated rat primary cortical neurons were log-transformed and autoscaled, mean and ± SEM values were calculated, and two-tailed paired *t* tests were performed (Extended Data Fig. 2-1). For graphical representation the data were back-transformed into the linear scale, error bars represent upper and lower limits of the back-transformed mean ± SEM. For analysis of transcript levels in time series of R6/1 mouse brain, data were log-transformed, mean and mean ± SEM were calculated for each group, and the average expression level of the respective transcript in eight-week-old wt animals was set as 0 (1 in linear scale). Generalized linear model with Gaussian distribution using the formula age + genotype + age:genotype was used, followed by Wald χ^2^ test (Type III test, performed with Car package in R; Fox and Weisberg, 2019) to determine *p* values of the coefficients. For graphical representation, data were back-transformed to the linear scale, with error bars showing back-transformed mean ± SEM.

For luciferase reporter assay, Firefly luciferase signals were normalized to the *Renilla* luciferase signal, data were then subjected to log transformation, mean centering, and autoscaling to obtain normal distribution. For graphical representation, the data were back-transformed to the original scale. One-way repeated-measures ANOVA with Greenhouse–Geisser correction followed by Tukey’s *post hoc* test was used to determine statistical significance compared with the full-length TCF4-B^–^ in different conditions using Prism 7 software (GraphPad; Extended Data Fig. 6-1).

Numbers of independent experiments and biological samples are indicated in figure legends; R6/1 mouse samples are summarized in Table 4.

**Table 4:** R6/1 mouse samples used for quantification of TCF4 mRNA and protein levels

		RT-qPCR				Western blotting		
Age	Genotype	CX	STR	HIP	CB	CX	STR	HIP
8 weeks	wt	8	8	8	8	6	7	7
	HD	7	6	7	7	6	7	7
12 weeks	wt	8	7	8	6	6	7	6
	HD	7	7	7	7	7	5	7
20 weeks	wt	7	7	7	7	7	5	6
	HD	6	6	5	5	6	6	7
30 weeks	wt	7	6	6	6	6	4	7
	HD	6	6	6	5	6	5	6

CX, cerebral cortex; STR, striatum; HIP, hippocampus; CB, cerebellum.

## Results

### Identification of TCF4 as a misregulated transcription factor in HD

**Figure 1.**
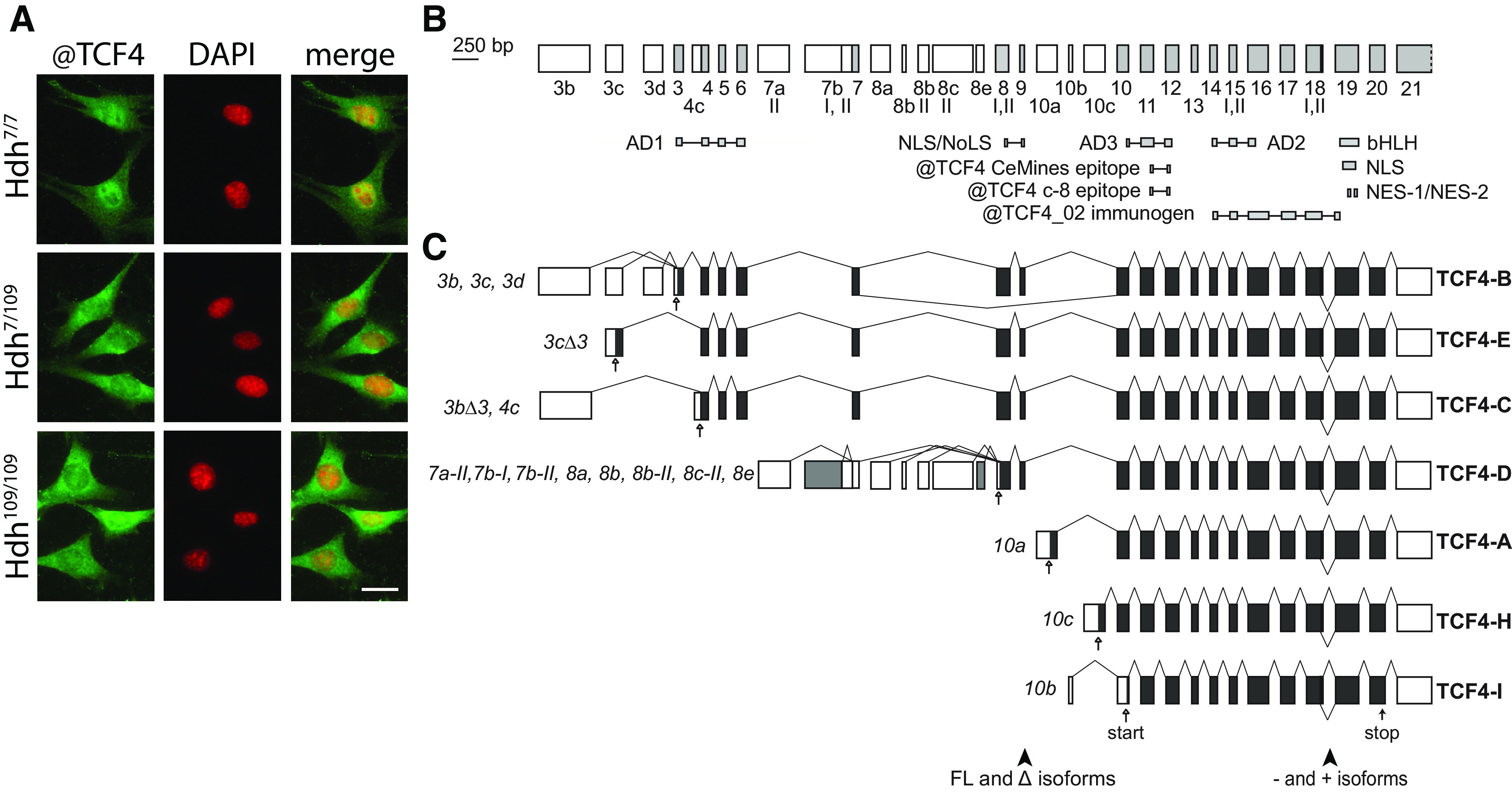
Differential immunocytochemical signal of endogenous TCF4 in Hdh cells and the structure and alternative splicing of mouse *Tcf4* gene. ***A***, Representative micrographs showing distribution of immunocytochemical signal obtained with anti-TCF4 antibodies (#HLH210, CeMines) in Hdh^7/7^, Hdh^7/109^, and Hdh^109/109^ cells. Antibodies used for the immunocytochemical screen in Hdh cells are listed in Extended Data Figure 1-1. Results of the screen with additional antibodies (CeMines) that showed differential signals in wt and mutant Hdh cells are shown in Extended Data Figure 1-2. DNA was counterstained with DAPI. Scale bar: 20 μm. ***B***, Mouse *Tcf4* genomic organization with exons drawn in scale. Gene structure layout and exon numbering is based on Sepp et al. (2011) for convenient comparison with human *TCF4* gene. 5′ exons are marked with white boxes and internal or 3′ exons are shaded in light gray. Exon names are displayed below boxes. Roman numerals designate alternative splice donor or acceptor sites, with some missing compared with the human *TCF4*. The regions encoding the respective domains of TCF4 and the epitopes of the used TCF4 antibodies [polyclonal @TCF4 (#HLH20, CeMines), polyclonal @TCF4_02 (Forrest et al., 2018), and monoclonal @TCF4 (Santa Cruz c-8)] are indicated below the gene structure. AD, activation domain; bHLH, basic helix-loop-helix; NLS, nuclear localization signal; NES, nuclear export signal; NoLS, nucleolar localization signal. ***C***, Alternative transcripts of mouse *Tcf4* grouped together according to the encoded TCF4 protein isoform. Translated and untranslated regions are designated as dark gray and white boxes, respectively. Transcripts are designated with the name of the 5′ exon and, where necessary, with the number of the splice site used in the 5′ exon. Accession numbers of the representative mouse *Tcf4* transcripts presented here are listed in Table 2. Transcripts using the 5′ exon *7b-I* (shown with gray) is translated into TCF4-D in mouse and TCF4-G in human and transcript *8e* (shown with gray) is unique to mouse. The name of the protein isoform is shown on the right. The position of the first in-frame start codon for each transcript and stop codon are shown with empty and filled arrows, respectively. Arrowheads at the bottom of the panel point to the regions of alternative splicing giving rise to Δ isoform or + and – isoforms.

Mislocalized nucleoporins and impaired nucleocytoplasmic transport have been described in several HD models and patients (Grima et al., 2017). We used a panel of >200 antibodies (Extended Data Fig. 1-1) generated against various transcription and transcription associated factors to screen for differential immunocytochemical signals in mouse striatal progenitor cell lines Hdh^7/7^ (wt), Hdh^7/109^ (heterozygous for HD mutation), and Hdh^109/109^ (homozygous for HD mutation). Our screen revealed 8 antibodies that showed differential signals in wt and mutant Hdh cells (Fig. 1*A*; Extended Data Fig. 1-2), including the FOXO3 antibodies that display increased nuclear signals in the mutant cells as reported earlier (Kannike et al., 2014). It must be noted, however, that most of the used antibodies have not been validated for immunocytochemical detection of endogenous proteins, and thus the results of the screen should be interpreted with caution. Antibodies generated against the basic helix-loop-helix (bHLH) transcription factor TCF4 showed reduced nuclear signals in Hdh cell lines expressing expanded *Htt* alleles (Fig. 1*A*). The specificity of the nuclear signal of these TCF4 antibodies has been demonstrated, whereas the cytoplasmic signal has been shown to be unspecific (Sepp et al., 2017). Therefore, our results suggest a difference in TCF4 localization and/or expression in Hdh^7/7^, Hdh^7/109^, and Hdh^109/109^ cells. Considering that TCF4 is an essential transcription factor in brain development and is linked to intellectual disability (Zweier et al., 2007; Kalscheuer et al., 2008; Blake et al., 2010), we sought to further characterize the potential misregulation of TCF4 in HD.

10.1523/ENEURO.0197-21.2021.f1-1 Extended Data Figure 1-1 List of antibodies used in immunocytochemical screen in Hdh cell line cells. *Catalogue numbers starting with sc refer to antibodies from Santa Cruz, all other antibodies are from CeMines. Download Figure 1-1, XLS file.

**Figure 2.**
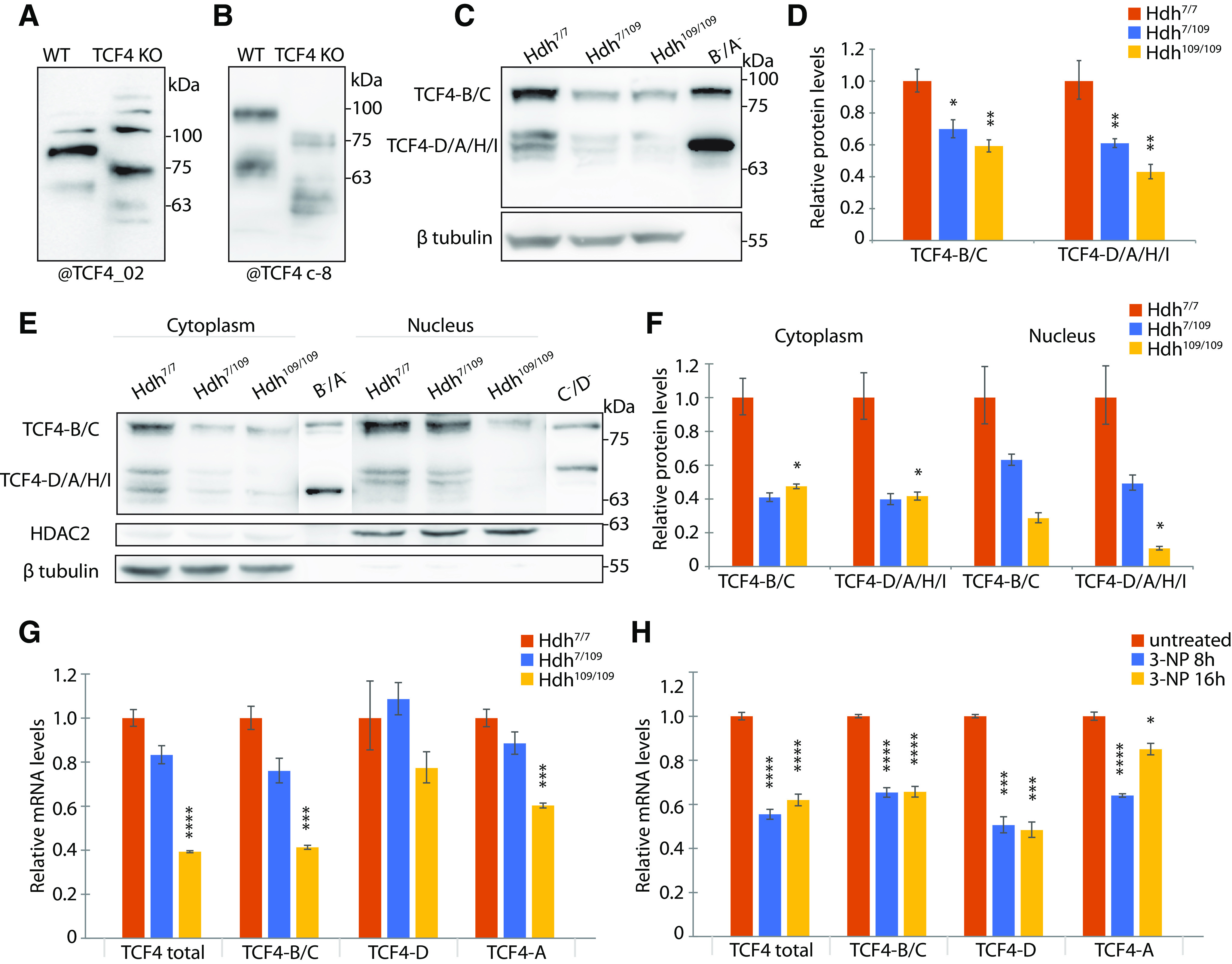
Protein and mRNA levels of transcription factor TCF4 in HD cell models. ***A***, ***B***, Validation of TCF4_02 polyclonal (***A***) and TCF4 c-8 monoclonal antibodies (***B***). Western blot analysis of E18 wt and *Tcf4* KO mice brain total lysates (Li et al., 2019). The KO mice express truncated TCF4 proteins that lack C terminus of the wt protein, but maintain the regions targeted by the used antibodies. ***C***, ***E***, Representative Western blot analysis of TCF4 protein levels in total lysates (***C***) and cytoplasmic and nuclear fractions (***E***; 100 and 50 μg of protein, respectively) of wt Hdh^7/7^, heterozygous mutant Hdh^7/109^ and homozygous mutant Hdh^109/109^ cells. *In vitro* translated human TCF4-A^–^, TCF4-B^–^, TCF4-C^–^, and TCF4-D^–^ were added as controls. β tubulin served as a loading control for total and cytoplasmic lysates and HDAC2 for nuclear lysates. ***D***, ***F***, Quantification of data in ***C*** (*n* = 8 independent experiments) and in ***E*** (*n* = 3), respectively. TCF4-B and TCF4-C were quantified together as high Mw isoforms (TCF4-B/C), and TCF4-D, TCF4-A^+^, and TCF4-A^–^ together as low Mw isoforms (TCF4-D/A^–^/A^+^). In ***D***, TCF4 signals were normalized to either the levels of β tubulin (for three experiments) or Coomassie Blue staining (for five experiments). In ***F***, signal intensities were normalized to β tubulin signal intensities in the cytosolic fraction and to HDAC2 in nuclear fraction. The relative TCF4 levels in Hdh^7/7^ were arbitrarily set as 1. The data were log-transformed, autoscaled, means and SEMs were calculated, and statistical significance is shown relative to the levels of respective TCF4 isoforms measured in Hdh^7/7^ cells; *p < 0.05, **p < 0.01, paired Student’s *t* test. Precise *p* values are reported in Extended Data Figure 2-1. The data were back-transformed into the linear scale for graphical representation, error bars represent upper and lower limits of back-transformed mean ± SEM. ***G***, ***H***, RT-qPCR analysis of total *Tcf4* mRNA, transcripts encoding long (TCF4-B and TCF4-C), medium (TCF4-D), and short (TCF4-A) isoforms in mouse Hdh cells (***G***; *n* = 3) and in rat primary cortical neurons untreated or treated with 3-NP for 8 or 16 h (***H***; *n* = 5). *Tcf4* mRNA levels were normalized to the levels of *Sdha* or *Hprt1* mRNA, respectively. The data were log-transformed, autoscaled, and the mean values and mean ± SEM were calculated. Statistical significance is shown relative to the expression level of the respective *Tcf4* transcripts in Hdh^7/7^ cells or untreated neurons. The data were back-transformed into linear scale for graphical depiction and error bars represent upper and lower limits of back-transformed mean ± SEM; *p < 0.05, ***p < 0.005, ****p < 0.001, paired Student’s *t* test (precise *p* values are reported in Extended Data Figure 2-1).

10.1523/ENEURO.0197-21.2021.f1-2 Extended Data Figure 1-2 Differential immunocytochemical signals obtained with unvalidated antibodies against different transcription factors in Hdh cells. Representative micrographs showing distribution of signals with antibodies (***A***) anti-Foxa (#FOX1, CeMines), (***B***) anti-Foxk2v1 (#FOX240, CeMines), (***C***) anti-Hmgn2 (#HMG50, CeMines), (***D***) anti-Isl1 (#LIM1, CeMines), (***E***) anti-Six2 (#SIX2, CeMines), and (***F***) anti-Trim21 (#ZF91, CeMines) in Hdh^7/7^, Hdh^7/109^, and Hdh^109/109^ cells. Anti-Foxa2, anti-Foxk2v1, anti-Isl1, anti-Six2 and anti-Trim21 showed distribution shifted towards the nucleus, and anti-Hmgn2 showed dots in the nucleus (arrows) in mutant Hdh cells compared to wt Hdh cells. DNA was counterstained with DAPI. Scale bar: 20 μm. Download Figure 1-2, EPS file.

Human *TCF4* is a complex gene with 41 exons, out of which 21 are alternative 5′ exons. Human TCF4 protein isoforms with 18 different N termini and additional variance resulting from alternative splicing at the cassette exons 8–9 (full-length and δ isoforms) and exon 18 [+/− isoforms differing by four amino acids (RSRS)] have been described (Sepp et al., 2011). To elucidate the structure of the mouse *Tcf4* gene we gathered data from public databases about mouse *Tcf4* expressed sequences (Fig. 1*B*,*C*; Table 2). The general gene structure of mouse *Tcf4* is similar to human *TCF4.* Compared with the human *TCF4* gene, fewer transcripts are reported for mouse *Tcf4* and these encode protein isoforms with seven different N termini (Fig. 1*B*). There were two rare TCF4-BΔ and one TCF4-E isoform encoding transcripts present in the databases. Our analysis of mouse *Tcf4* data revealed seven alternative TCF4-D-coding transcripts linked to different 5′ untranslated exons with translation initiation codon in exon 8 for all of them. Of note, a nucleotide addition in 5′ alternative exon 7b-I prevents the expression of TCF4-G in mice and TCF4-D is coded instead (Fig. 1*C*). The transcript starting from exon 8d, expressed in human, has not been described in mouse. Instead, transcript 8e (with a different transcription start site) is expressed in mouse.

Collectively, we have determined that the *TCF4* gene structure is highly conserved between mouse and human and our results suggest misregulation of TCF4 in the mutant HTT-expressing mouse striatal cells.

### TCF4 levels are reduced in cell-based HD models

To dissect the contribution of different TCF4 isoforms to the altered immunocytochemical signal of TCF4 detected in HD cells, we next studied TCF4 protein levels in Hdh cell lines by Western blotting. First, we used cortical lysates from *Tcf4* KO mice (Li et al., 2019) to validate the specificity of two TCF4 antibodies, polyclonal antibody anti-TCF4_02 (Forrest et al., 2018) and a commercial monoclonal antibody anti-TCF4 c-8 (Santa Cruz; Fig. 2*A*,*B*) and determined the mobility pattern of different TCF4 isoforms overexpressed in HEK293 cell lysates (data not shown). We then analyzed total cell lysates of Hdh cells by Western blotting with polyclonal anti-TCF4_02 antibody (*n* = 3; Fig. 2*C*) and anti-TCF4 c-8 antibody (*n* = 5). Based on the predicted molecular weight (Mw) and mobility of *in vitro* translated isoforms TCF4-B^–^ and TCF4-A^–^, we divided the detected TCF4 protein bands into two groups: high Mw isoforms, similar to isoforms TCF4-B and TCF4-C and their respective +/− isoforms, and medium/low Mw isoforms, which probably correspond to TCF4-D, TCF4-A, TCF4-H, and TCF4-I and their respective +/− isoforms. Of note, + isoforms have higher mobility than isoforms in SDS-PAGE gels as described previously (Sepp et al., 2011). We observed a significant >30% decrease of both high and low Mw TCF4 isoforms in both mutant Hdh cell lines compared with wt Hdh cells (Fig. 2*C*,*D*).

10.1523/ENEURO.0197-21.2021.f2-1 Extended Data Figure 2-1 Statistical tests used and *p* values for Figure 2. Download Figure 2-1, XLS file.

Next, to further decipher the changes in TCF4 levels we fractionated Hdh cells into cytoplasmic and nuclear lysates and analyzed the lysates by Western blotting with polyclonal TCF4_02 antibody (*n* = 3; Fig. 2*E*). *In vitro* translated isoforms TCF4-B^–^, TCF4-A^–^, TCF4-C^–^, and TCF4-D^–^ were used to decipher bands detected in Hdh cell lysates. TCF4 protein levels were reduced in both cytoplasmic and nuclear fractions of mutant Hdh cells (Fig. 2*F*). In mutant Hdh cell cytoplasm the levels of TCF4 were downregulated to ∼40% of TCF4 detected in wt cells, with statistical significance only for Hdh^109/109^ (Fig. 2*F*). In the nuclear fraction, a visible reduction was observed in both mutant cell lines with all TCF4 isoforms, albeit statistical significance was reached only for TCF4 D/A/H/I where protein levels in Hdh^109/109^ cells were reduced to 10% of the levels of these isoforms in wt cells (Fig. 2*F*). Taken together, TCF4 protein levels were reduced in both fractions and especially dramatically in Hdh^109/109^ nuclear fraction. Interestingly, distinct set of medium/low Mw TCF4 isoforms were present in the nucleus compared with cytoplasm, although the isoform patterning did not differ between Hdh genotypes (Fig. 2*F*).

To further elucidate changes of TCF4 in HD we sought to study mRNA levels of total *Tcf4*, combination of the longer transcripts encoding TCF4-B/C and transcripts encoding the most abundant shorter protein isoforms TCF4-D and TCF4-A in mouse Hdh cell lines (*n* = 3; Fig. 2*G*). Total *Tcf4* mRNA levels were pronouncedly downregulated in mutant homozygous Hdh cells – to 40% of total *Tcf4* levels in wt Hdh cells. Similarly, transcripts encoding isoforms TCF4-B and TCF4-C were reduced to 30%, and transcripts encoding TCF4-A to 40% in Hdh^109/109^ cells in comparison with wt cells. TCF4-D-encoding mRNAs are transcribed from seven alternative 5′ exons out of which we analyzed transcripts with *8c-II* 5’exon (Fig. 1*C*). No significant change in TCF4-D-encoding *8c-II* transcript levels were detected (Fig. 2*G*).

To validate our findings from genetic HD model cell lines, we studied *Tcf4* mRNA levels in an induced chemical model, cultured neurons treated with mitochondrial toxin 3-NP. We treated rat cortical neuron cultures with 0.5 mm 3-NP at DIV6–DIV8 for 0–16 h to mimic HD (*n* = 5). We determined the same *Tcf4* transcripts as in Hdh cells. Total *Tcf4* mRNA levels decreased in cultured cortical neurons after 8- and 16-h treatment with 3-NP to 60% of *Tcf4* levels measured in untreated neurons (Fig. 2*H*). We observed reduction of TCF4-B and TCF4-C encoding mRNAs in response to 3-NP treatment. After 8- or 16-h exposure to 3-NP the levels of TCF4-A encoding mRNAs were decreased to 64% or to 85% of the levels detected in untreated neurons, respectively. Again, we measured levels of *8c-II* transcript as a representative of transcripts encoding TCF4-D and observed a 50% decrease following 3-NP treatment.

Taken together, the levels of *Tcf4* transcripts are reduced in cortical neurons treated with 3-NP as well as in HD striatal cell lines. This is consistent with the reduced protein levels of TCF4 in HD striatal cells.

### Differential downregulation of *Tcf4* transcripts in R6/1 mouse brain

Progressive neurodegeneration in HD is most prevalent in striatum and cerebral cortex, but other brain structures and circuits may also be affected (McColgan and Tabrizi, 2018). To extend our finding in HD cells to animal models, we decided to study *Tcf4* mRNA levels in the transgenic R6/1 mouse model of HD, where N-terminal exon 1-containing fragment of human mutant huntingtin with 115 CAG repeats is expressed. We analyzed mRNA levels in the striatum, cortex, hippocampus and cerebellum of wt and R6/1 mouse at 8, 12, 20, and 30 weeks of age.

**Figure 3.**
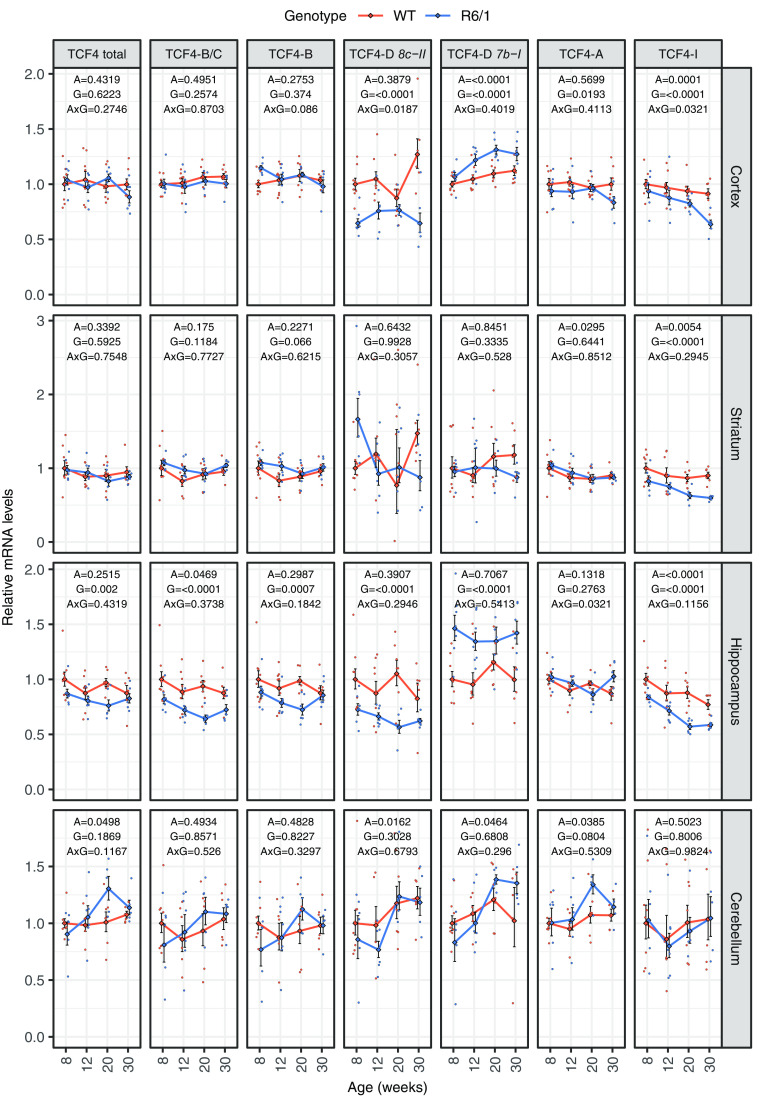
*Tcf4* mRNA levels in R6/1 mouse brain. RT-qPCR analysis of mRNA levels of total *Tcf4* and selected *Tcf4* transcripts encoding alternative TCF4 isoforms. RT-qPCR analysis was conducted in cortex, striatum, hippocampus, and cerebellum from wt and R6/1 mice at four ages (8, 12, 20, and 30 weeks of age). Number of samples included in each experiment is indicated in Table 4. The transcripts encoding long isoforms TCF4-B and TCF4-C were measured together (TCF4-B/C) and separately in the case of TCF4-B; two TCF4-D encoding transcripts *8c-II*, and *7b-I* as well as transcripts encoding short isoforms TCF4-A, and TCF4-I were quantified. *Tcf4* mRNA levels were normalized to geometric mean of the levels of *Hprt1*, *Tbp*, and *Gapdh*. The average expression level of respective transcript in eight-week-old wt animals was set as 1. The average expression of the respective transcripts in respective groups is shown with lines, error bars indicate SEM, data from all individual animals are shown with dots. Generalized linear model using the formula age + genotype + age:genotype was used, followed by Wald χ^2^ test to determine *p* values of the coefficients (shown at the top of each graph). A, age; G, genotype; A×G, interaction between age and genotype.

To study in detail *Tcf4* mRNA expression in R6/1 mouse brain we quantified mRNA levels of total *Tcf4*, combination of the longer transcripts encoding TCF4-B and TCF4-C (TCF4-B/C), transcripts encoding only TCF4-B, and the most prevalent shorter transcripts encoding TCF4-D, TCF4-A, and TCF4-I by RT-qPCR (Fig. 3). There was no change in the total levels of *Tcf4* mRNAs in the cerebral cortex, striatum and cerebellum, whereas downregulation of total *Tcf4* expression was seen in the hippocampus of R6/1 mice when compared with wt mice at all the ages analyzed. More changes were detected in R6/1 mouse brain at the level of alternative transcripts with decreased longer transcripts encoding TCF4-B and TCF4-B/C in the hippocampus, transcripts encoding TCF4-A in the cerebral cortex and transcripts encoding TCF4-I in the cerebral cortex, hippocampus and striatum. Out of the studied TCF4-D encoding transcripts the expression of *8c-II* mRNA was downregulated and *7b-I* upregulated in the cerebral cortex and hippocampus of mutant versus wt mice. To sum up, we observed a reduction in total *Tcf4* mRNA levels in R6/1 mouse hippocampus, and a comprehensive study revealed an intriguing variability and specific upregulations and downregulations of certain *Tcf4* transcripts already before the onset of HD symptoms. Changes at the level of alternative *Tcf4* transcripts in the cerebral cortex and striatum, and no differences in the *Tcf4* expression in the cerebellum were observed over a time course, from 8 to 30 weeks of age, in R6/1 compared with wt mouse.

### TCF4 protein levels are decreased in the hippocampus of R6/1 mouse

**Figure 4.**
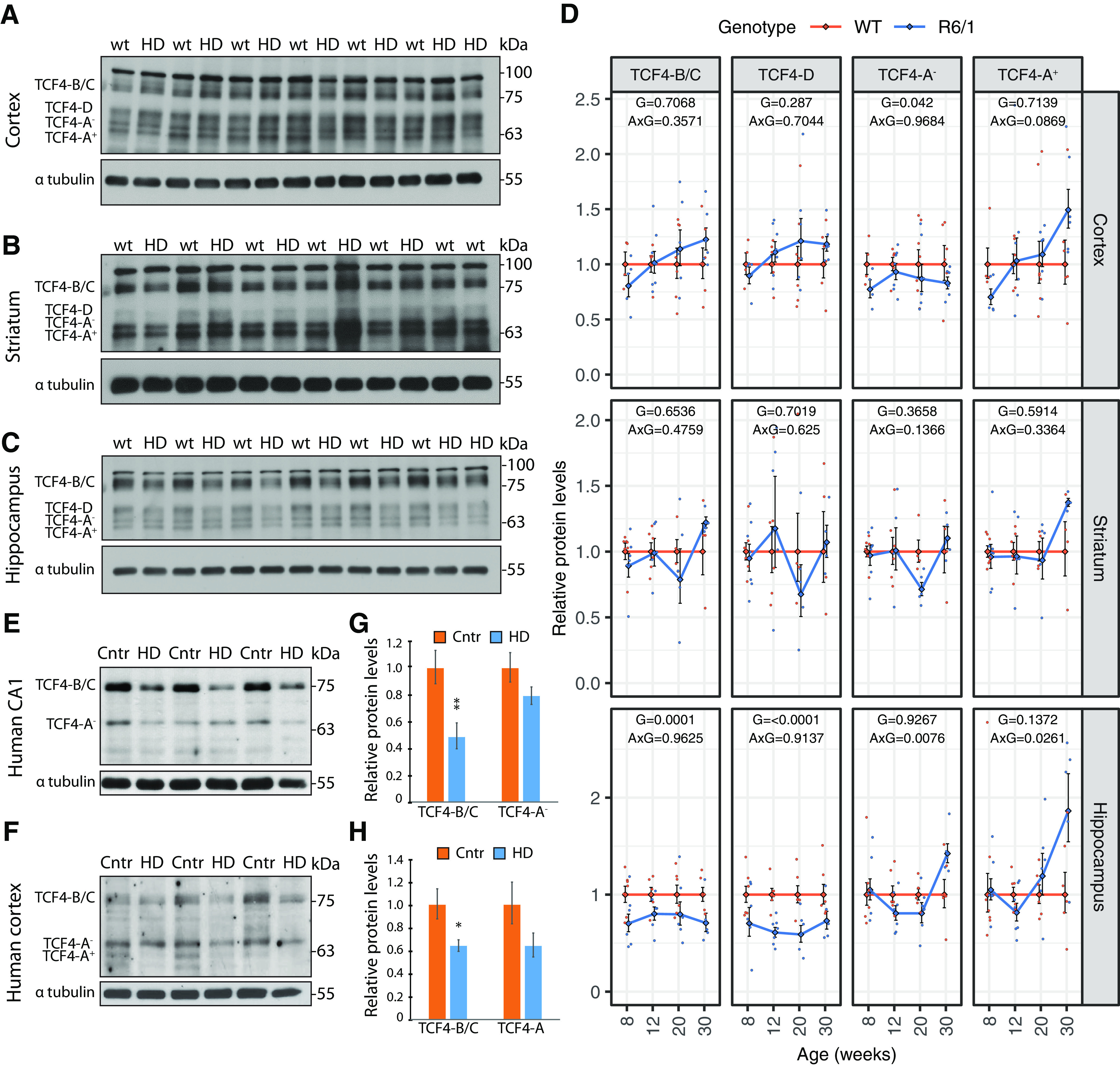
TCF4 protein levels in different brain regions of R6/1 mouse and HD patients. ***A–C***, Western blot analysis of TCF4 protein levels in wt and R6/1 mouse cerebral cortex (***A***), striatum (***B***), and hippocampus (***C***) at 12 weeks of age (blots not shown for 8, 20, and 30 weeks of age). α tubulin served as loading control. ***D***, Quantifications of Western blotting data. Number of samples included in each experiment is indicated in Table 4. The average expression level of the respective isoform in wt animals of each age group was set as 1. The average expression of the respective isoform in respective groups is shown with lines, error bars indicate SEM, data from all individual animals are shown with dots. Generalized linear model using the formula genotype + age:genotype was used, followed by Wald χ^2^ test to determine *p* values of the coefficients (shown at the top of each graph), with G designating genotype, and A×G designating interaction between age and genotype. ***E***, ***F***, Representative Western blot analysis of TCF4 protein levels in human hippocampal CA1 region and cerebral cortex, respectively, of healthy controls (Cntr) and HD patients (HD). α tubulin served as loading control. Human postmortem samples are described in Table 1. ***G***, ***H***, Quantification of data in ***E***, ***F***, respectively, and additional Western blottings (data not shown). For human hippocampal CA1 region a total of seven healthy controls and 10 HD patients were analyzed, and for human cortex, six healthy controls and six HD patients were analyzed. TCF4 signal intensities were normalized to α tubulin signal intensities. The relative TCF4 levels in healthy controls were arbitrarily set as 1. Data were log-transformed, means and SEMs were calculated, and two-tailed two-sample equal variance *t* test was used. Data were back-transformed into the linear scale for graphical representation, error bars represent upper and lower limits of back-transformed mean ± SEM; *p < 0.05, **p < 0.01; precise *p* values are stated in Extended Data Figure 4-1.

We next asked whether the changes detected in *Tcf4* mRNA expression were reflected in the levels of TCF4 protein isoforms in R6/1 mouse brain. We analyzed TCF4 protein levels by Western blotting with monoclonal TCF4 antibody c-8 in the striatum, cortex and hippocampus in the same mice where *Tcf4* mRNA levels were analyzed. The quality of Western blottings enabled quantification of the separate TCF4 bands that were assigned as potential B/C, D, A^–^, and A^+^ isoforms in the order of increasing mobility in PAGE based on theoretical Mw and comparisons with *in vitro* translated TCF4 isoforms (Fig. 2*C*,*E*). No band corresponding to TCF4-I was detected. The patterns of TCF4 isoforms expressed in the two pallial structures, cerebral cortex and hippocampus, were similar, whereas in the subpallial striatum the relative proportions of TCF4-D were lower (Fig. 4*B*,*D*). Comparisons of TCF4 isoform levels across wt and transgenic mice revealed decreased levels of TCF4-A^–^ in the R6/1 mouse cerebral cortex and a reduction of TCF4-B/C and TCF4-D by 30% and 40%, respectively, in R6/1 mouse hippocampus at all the ages analyzed (Fig. 4*D*). No statistically significant differences were detected in the striatum. Collectively, the results of Western blot analysis in R6/1 mouse brain are in good agreement with the major effects seen for *Tcf4* mRNA levels, further indicating that the changes in TCF4 are isoform and brain region specific.

10.1523/ENEURO.0197-21.2021.f4-1 Extended Data Figure 4-1 Statistical test used and *p* values for Figure 4. Download Figure 4-1, XLS file.

### High Mw isoforms of TCF4 are decreased in HD patient’s hippocampus

We observed downregulation of several *Tcf4* transcripts and protein isoforms in R6/1 mouse hippocampus and cerebral cortex, therefore we next analyzed TCF4 protein levels in these regions of HD patients. Tissue lysates of hippocampal CA1 region and cerebral cortex from postmortem HD patients and neurologically healthy controls were analyzed by Western blotting using monoclonal anti-TCF4 antibody c-8. The expression pattern of TCF4 protein isoforms in human brain tissues differed from mouse, although bands likely corresponding to TCF4-B/C and TCF4-A could be detected (Fig. 4*E*,*F*). TCF4 high Mw isoforms TCF4-B/C were significantly reduced in the hippocampus of HD patients (Fig. 4*E*,*G*), while the expression of shorter isoforms, likely TCF4-A^–^, was not significantly changed (Fig. 4*G*). We detected statistically significant reduction of TCF4-B/C in the cerebral cortex of HD patients and decreased levels of TCF4-A (sum of TCF4-A^–^ and TCF4-A^+^) that did not reach to statistical significance (Fig. 4*F*,*H*). Decreased TCF4 levels in human hippocampus and cerebral cortex corroborate our findings in R6/1 mouse, suggesting dysregulation of TCF4 expression in HD hippocampus and cortex in both mice and humans.

### Differential expression of BDNF and TCF4 dimerization partners in R6/1 mouse brain

**Figure 5.**
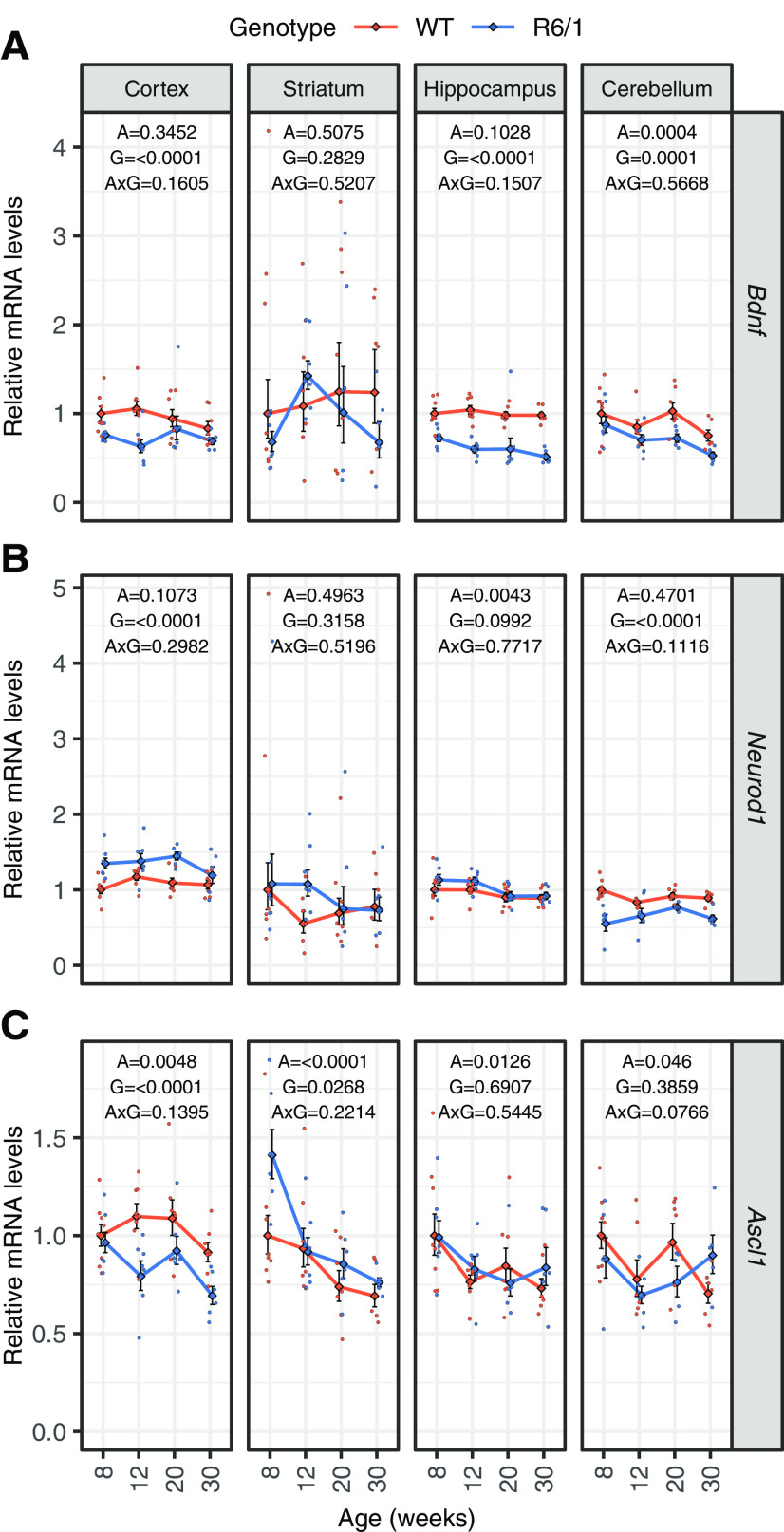
Expression levels of mRNAs encoding BDNF and TCF4 dimerization partners ASCL1 and NEUROD1 in R6/1 mouse brain. ***A*–*C***, RT-qPCR analysis of *Bdnf* (***A***), *Neurod1* (***B***), and *Ascl1* (***C***) transcripts in wt and R6/1 mouse brain regions at 8, 12, 20, or 30 weeks of age from the same samples used for the analysis of *Tcf4* transcript levels. Number of samples included in each experiment is indicated in Table 4. *Bdnf*, *Neurod1*, and *Ascl1* mRNA levels were normalized to the geometric mean of the levels of *Hprt1*, *Tbp*, and *Gapdh* mRNA. The average expression level of respective transcript in eight-week-old wt animals was set as 1. The average expression of the transcripts in respective groups is shown with lines, error bars indicate SEM, data from all individual animals are shown with dots. Generalized linear model using the formula age + genotype + age:genotype was used, followed by Wald χ^2^ test to determine *p* values of the coefficients (shown at the top of each graph). A, age; G, genotype; A×G, interaction between age and genotype.

The reduction of wt HTT and expression of mutant HTT have been shown to decrease the levels of the neurotrophic factor BDNF (Zuccato et al., 2001), and this decrease has been observed in many HD models as well as in HD patients (Ferrer et al., 2000; Duan et al., 2003; Zhang et al., 2003). It has also been reported that TCF4 regulates BDNF expression through an enhancer region (Tuvikene et al., 2021). To this end, we quantified *Bdnf* mRNA levels in the R6/1 different brain regions (Fig. 5*A*). Our analysis revealed that the presence of mutant HTT significantly affects the expression of *Bdnf* gene in R6/1 mouse cerebral cortex, hippocampus and cerebellum. No change of *Bdnf* mRNA levels was detected in R6/1 mouse striatum. Moreover, the expression level of *Bdnf* mRNA was very low in the striatum compared with other brain regions studied, which is in line with the knowledge that *Bdnf* expression is very limited in the striatum and most of striatal BDNF protein is anterogradely transported there from the cortex (Altar et al., 1997). Collectively, *Bdnf* mRNA levels were reduced in the brain of R6/1 mouse used in this study, possibly partially because of decreased TCF4 levels in this model system.

Transcription factor TCF4 belongs to E-protein family of bHLH transcription factor superfamily that bind E-box DNA sequence as homodimers or heterodimers. TCF4 is one of the three E-protein partners for proneural transcription factor ASCL1 and neuronal differentiation factor NEUROD1 (Massari and Murre, 2000; Dennis et al., 2019) that display altered gene expression in HD iPSC cells (The HD iPSC Consortium, 2017). Therefore, we determined mRNA levels of these dimerization partners in the R6/1 mouse brain regions. While the biggest decrease of many *Tcf4* mRNAs were detected in hippocampus of R6/1 mouse, mRNA levels of *Neurod1* and *Ascl1* were not affected in this brain region (Fig. 5*B*,*C*). Along with downregulation of several short *Tcf4* transcripts, the levels of *Ascl1* mRNA were reduced (Fig. 5*B*), whereas *Neurod1* mRNA was upregulated in R6/1 mouse cerebral cortex when compared with wt mice (Fig. 5*B*). Unchanged mRNA levels of *Neurod1* in the striatum and downregulated levels in the cerebellum were paralleled with almost unchanged mRNA levels of *Tcf4* in these regions (Fig. 5*B*). Additionally, the mRNA levels of *Ascl1* were increased in the striatum of R6/1 mouse in comparison to wt mice (Fig. 5*C*), whereas there was no significant change of *Ascl1* mRNA levels in the cerebellum (Fig. 5*C*). Collectively, the analysis of the two TCF4 dimerization partners indicate that their expression is also dysregulated in the R6/1 HD mouse model, although in a different regional pattern compared with TCF4, implying a wide-spread dysregulation of E-box-dependent transcription, even in brain regions where TCF4 was not affected.

### TCF4 and ASCL1 synergistically transactivate reporter gene transcription in rat cortical and hippocampal neurons

**Figure 6.**
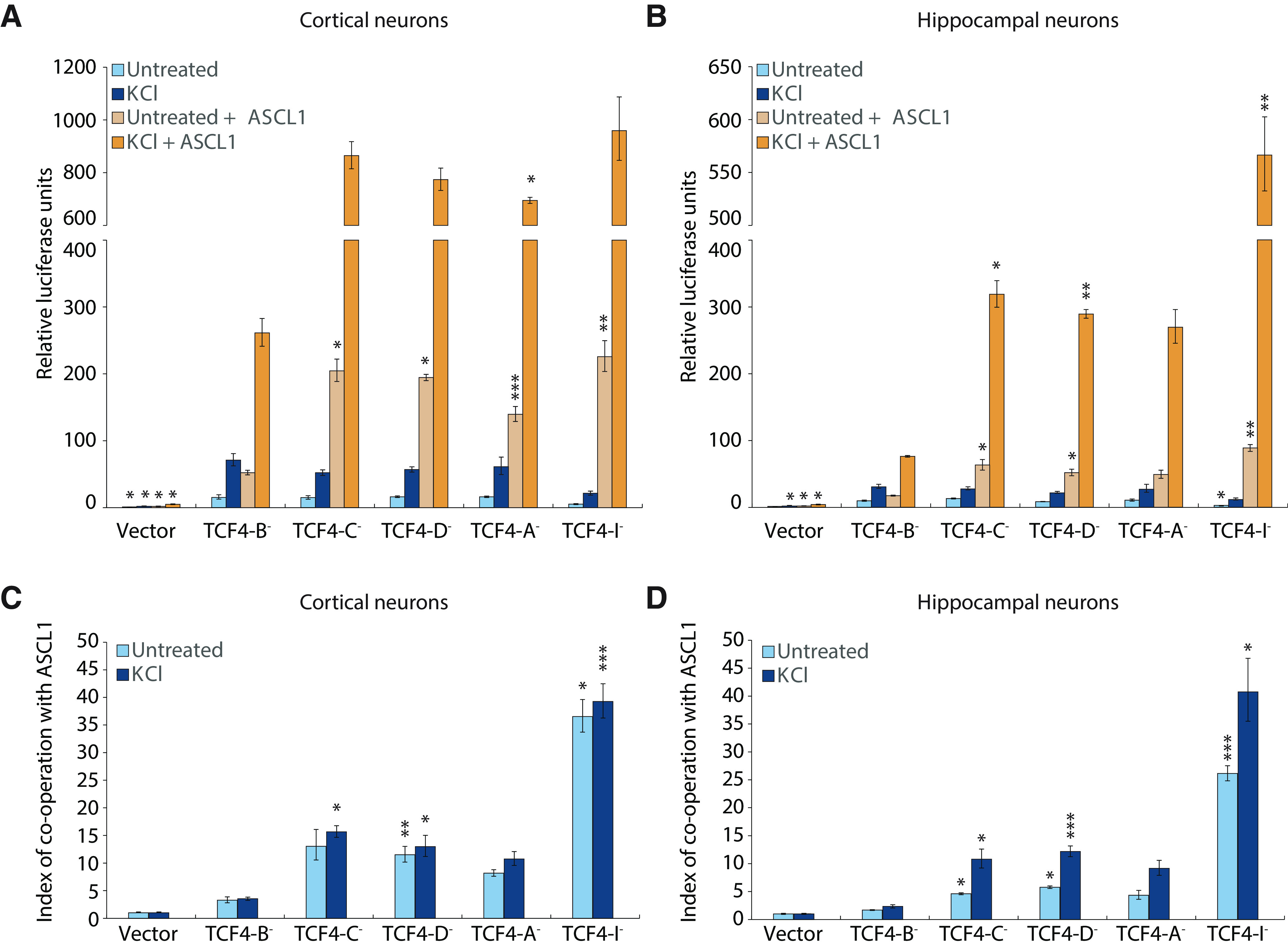
Differential co-operation of TCF4 isoforms with ACSL1 in cortical and hippocampal neurons. ***A***, ***B***, Specific TCF4 isoforms and ASCL1 under the control of *EF1a* and *PGK1* promoter, respectively, were overexpressed in rat primary cortical (***A***) or hippocampal (***B***) neurons at 6 DIV. For luciferase reporter assay, the cells were co-transfected with firefly luciferase reporter construct with 12 μE5 E-boxes (CACCTG) in front of *TK* promoter, and for normalization *Renilla* luciferase construct with *PGK1* promoter was used. One day after transfection, neurons were treated with 25 mm KCl for 8 h or left untreated. Luciferase signals from three independent experiments were measured in duplicates, normalized to *Renilla* signals, log-transformed, mean-centered, and autoscaled for statistical analysis. Data were back-transformed to original scale and are presented relative to the signals measured from empty vector-transfected (Vector) untreated cells (arbitrarily set 1). ***C***, ***D***, Index of co-operation between TCF4 isoforms and ASCL1 in basal and depolarized conditions calculated from data in ***A*** or ***B***, respectively. Normalized luciferase data were used for calculating co-operation indices, data were log-transformed, mean-centered and autoscaled for statistical analysis and back-transformed for graphical depiction. The index value of 1 implies simple summation, while values above 1 indicate synergism and below 1 antagonism. The co-operation is expressed separately for untreated or KCl-treated cells. ***A–D***, Error bars indicate SEM. One-way ANOVA followed by Tukey’s *post hoc* test was used for statistical analysis (precise *p* values are reported in Extended Data Figure 6-1). Asterisks show statistical significance relative to cells overexpressing TCF4-B¯ in basal conditions (***A***, ***B***). For cooperation indexes, statistical significance is shown within untreated or KCl-treated cells relative to TCF4-B¯ in the respective condition (***C***, ***D***; precise *p* values are reported in Extended Data Figure 6-1); *p < 0.05, **p < 0.01, ***p < 0.001.

Different transactivation capacity of specific TCF4 protein isoforms has been reported in HEK293 cells and in mixed culture of rat cortical and hippocampal neurons (Sepp et al., 2011, 2012, 2017). Additionally, TCF4-A^–^ activates *Gadd45g* promoter in co-operation with ASCL1 in unstimulated and KCl-depolarized neurons (Sepp et al., 2017). Here, we observed notable cortex and hippocampus-specific differences in the expression of different TCF4 isoforms and *Ascl1* between wt and R6/1 mouse. Therefore, we set out to comprehensively study the transactivation capability of major TCF4 protein isoforms and their synergism with dimerization partner ASCL1 separately in hippocampal and cortical neurons before and after induction of neuronal activity. For this, we transfected neurons with E-box-dependent luciferase reporter, together with different TCF4 isoform-encoding and ASCL1-encoding constructs. To mimic neuronal activity, cells were chronically depolarized using KCl. In unstimulated cells overexpressing TCF4 alone, all TCF4 protein isoforms induced reporter gene expression to relatively similar extent, except TCF4-I^–^ that induced reporter gene expression three times less compared with full-length isoform TCF4-B^–^ in both cortical and hippocampal neurons (Fig. 6*A*,*B*). KCl treatment induced TCF4-dependent transcription on average 2-fold over basal activity levels of the same TCF4 isoform, with no major differences between TCF4 isoforms. In contrast, co-expression of TCF4 and ASCL1 revealed differential upregulation of reporter activity (Fig. 6*A*,*B*). TCF4-B^–^ showed minimal increase of reporter activation in co-operation with ASCL1, whereas ∼20-fold increase over TCF4-I^–^ alone was seen when TCF4-I^–^ and ASCL1 were expressed together. We also noted that reporter activity was higher when co-expressing ASCL1 with different TCF4 isoforms in cortical neurons compared with hippocampal neurons. The reporter gene expression was further induced by KCl treatment in cells co-expressing TCF4 and ASCL1. However, the total reporter gene expression level was drastically lower in TCF4-B^–^ overexpressing neurons compared with other TCF4 isoforms under the same conditions. The highest reporter expression was seen with the shortest TCF4 isoform TCF4-I^–^ co-expressed with ASCL1 and treated with KCl, where its low initial transactivation capacity was more than fully compensated. To analyze transcriptional synergy between TCF4 and ASCL1, we calculated co-operation indices according to Chang et al. (1996). All TCF4 isoforms had a synergistic effect with ASCL1 in both cortical and hippocampal neurons (6C and 6D). The co-operation was the smallest for TCF4-B^–^, equally high for TCF4-C^–^, TCF4-D^–^, and TCF4-A^–^, and exceptionally high for TCF4-I^–^. Additionally, KCl treatment increased the synergism between TCF4 isoforms and ASCL1 in hippocampal neurons, but not in cortical neurons. To conclude, these results illustrate the TCF4 isoform-dependent differential transactivation in neurons and suggest brain region specific TCF4-dependent gene transcription. Furthermore, considering the brain region-specific dysregulation of TCF4 and its binding partners in HD, this differential synergism between TCF4 and its binding partners could also play a role in the etiology of HD.

10.1523/ENEURO.0197-21.2021.f6-1 Extended Data Figure 6-1 Statistical tests used and *p* values for Figure 6. Download Figure 6-1, XLS file.

## Discussion

An increasing body of evidence suggests that HD is not only a neurodegenerative disease but also has a strong neurodevelopmental component (The HD iPSC Consortium, 2017; Siebzehnrübl et al., 2018; Barnat et al., 2020). Here, we set out to study TCF4 in the context of HD. Haploinsufficiency of TCF4 causes a rare neurodevelopmental Pitt-Hopkins syndrome, TCF4 has been linked to schizophrenia and mild to moderate intellectual disability, and has been shown to regulate neurogenesis, synaptic plasticity, memory and DNA methylation (Zweier et al., 2007; Blake et al., 2010; Kennedy et al., 2016; Jung et al., 2018; Page et al., 2018; Li et al., 2019). Here, we demonstrate that the expression of TCF4 is dysregulated in both cell-based and animal models of HD and in HD patients.

TCF4 can regulate target gene expression both as homodimers and as heterodimers with class II proneural bHLH proteins such as ASCL1 and NEUROD1. ASCL1 is considered to have several functions in neurogenesis, for example it is important in preserving the pool of nerve cell progenitors, whereas NEUROD1 plays important roles in neuronal and glial differentiation and maturation (for review, see Dennis et al., 2019). In R6/1 mouse cerebral cortex we found that *Neurod1* mRNA levels were increased, *Ascl1* mRNA levels were decreased, and total levels of *Tcf4* transcripts remained unchanged. Additionally, in the cerebral cortex we detected decreased TCF4-A^–^ protein in R6/1 mice and TCF4-B/C proteins in HD patients. These detected changes of transcription factors might explain why the developing cerebral cortex of mutant HTT-expressing human fetuses present diminishing pool of proliferating cells and neural progenitors enter prematurely neuronal lineage specification (Barnat et al., 2020). We also found disease-dependent downregulation of *Neurod1* in the cerebellum and no significant expression change in the hippocampus of R6/1 mice, in combination with unchanged or drastically decreased *Tcf4* mRNA levels in cerebellum and hippocampus, respectively. NEUROD1 has functions in granule cells of these two brain regions (Miyata et al., 1999; Pleasure et al., 2000; Gao et al., 2009), and co-expression of NEUROD1 and TCF4 has been demonstrated in adult mouse DG immature granule neurons (Jung et al., 2018). Moreover, decreased protein levels of NEUROD1 were reported to affect neurogenesis in the adult hippocampus of the R6/2 mice, a fast-progressing HD model (Fedele et al., 2011). The study of HD patient-derived iPSC induced into mixed neural phenotypes showed that one-third of gene changes were in pathways regulating neuronal development and maturation, and downregulation of *NEUROD1* and *ASCL1* were also reported (The HD iPSC Consortium, 2017). Taken together, we showed HD-dependent brain region-specific altered expression levels of proneural *Ascl1* and *Neurod1* in adult mice with known relevant functions in these brain tissues. Delineating the expression of these transcription factors at earlier developmental stages of R6/1 mice could be of interest for future studies.

The human *TCF4* gene structure has been described by Sepp et al. (2011), and here we report a detailed description of mouse *Tcf4* gene structure and the numerous transcripts it encodes. The gene structure of *TCF4* is conserved between species suggesting functional relevance of potential protein isoforms. In general, the functions of alternative TCF4 isoforms are poorly studied and the reason for multitude of 5´exons with specific promoters, isoforms with N termini of different length, and +/− isoforms of TCF4 is still a puzzle. One of the early studies of TCF4 (known previously as E2-2, SEF2-1, and ITF2) revealed that TCF4-D inhibits MYOD (Skerjanc et al., 1996), and another study showed specific inhibition of brain-specific *FGF1B* by TCF4-B^–^, whereas TCF4-B^+^ did not have any effect (Liu et al., 1998). Furthermore, differential expression was shown for *Tcf4-A* and *Tcf4-B* in immune cell types, and isoform-specific regulation was shown for normal plasmacytoid dendritic cell development (Grajkowska et al., 2017). Recently, discrepant effects of TCF4 isoforms B and A were shown in the context of oligodendrocytes differentiation, and the authors propose that the differentiation promoting effect of TCF4 is specific to the long isoform (Wedel et al., 2020). Additionally, alternative TCF4 isoforms have differential transactivation potential in HEK293 cells and also in unstimulated and depolarized neurons (Sepp et al., 2011, 2017). Here, we report isoform-dependent co-operation of TCF4 with ASCL1 in neurons. Although functional dimerization of TCF4 and ASCL1 has been shown previously (Persson et al., 2000; de Pontual et al., 2009; Sepp et al., 2017), there is limited knowledge of their co-operation to activate transcription in neurons, specifically in cortical and hippocampal neurons. We found that there is a synergistic effect between all studied TCF4 protein isoforms and ASCL1, although extent of the synergy varies several times depending on the TCF4 protein isoform. For example, the low transactivation capability of TCF4-I^–^ overexpressed alone (probably mainly homodimers) is compensated when heterodimerized with ASCL1, whereas full-length TCF4-B^–^ shows limited synergism with ASCL1. Furthermore, we saw a depolarization-dependent increase in TCF4-ASCL1 synergy in hippocampal but not in cortical neurons. This suggests that the neuronal activity-dependent transcriptional regulation of TCF4 and its heterodimers could be fundamentally different in different brain regions and neuron populations. This could be relevant for developing alleviating therapies for diseases and disorders with disturbances of distinct TCF4 isoforms in specific brain regions affected as we have reported here for HD.

Highly similar total *TCF4* expression patterns have been reported for humans, rhesus monkeys, and mice at total *TCF4* level (Jung et al., 2018). TCF4 is involved in the early stages of neuronal development and highly expressed across the entire brain (Kim et al., 2020). In contrast, in adult brain the high expression of TCF4 is restricted to the pallial region and cerebellum (Kim et al., 2020). Here, we observed brain region-specific differences in the proportions of TCF4 isoforms expressed in mice, and our Western blot analysis of human TCF4 in the hippocampus and cerebral cortex of elderly adults showed a distinctive pattern of TCF4 isoforms divergent from that seen in adult mice. Importantly, we have validated all used TCF4 antibodies in *Tcf4* KO mice embryonic cortical tissue lysates, therefore we believe that bands detected on human immunoblot are specific. Additionally, we validated TCF4 antibodies with several *in vitro* translated human TCF4 isoforms, however, we cannot completely rule out the possibility of masking TCF4 isoform specific signals by other proteins with similar apparent Mw in Western blotting using human tissue lysates. Nevertheless, differential subcellular and brain region-specific expression of TCF4 isoforms suggests fine-tuning of expression, likely required for precisely controlled target gene expression. In our RT-qPCR study of *Tcf4* transcripts in R6/1 mice, we observed opposite change in the levels of TCF4-D encoding *8c-II* and *7b-I* mRNAs. This additional layer of complexity entails potential to differentially regulate multitude of 5′ alternative *TCF4* transcripts that eventually translate into the same TCF4 isoform. Our results indicate that the possibility of specific regulation is not a mere opportunity, but these transcripts actually are expressed differentially in R6/1 mouse hippocampus and cortex.

The most notable result of our study is the decreased levels of TCF4, a neurodevelopmentally important transcription factor, in the hippocampus and cerebral cortex of both HD mouse model and HD patients. For a long time, the role of hippocampus in HD has been overlooked. However, recently its role in HD has been acknowledged as symptoms such as decline of cognitive functions and problems with learning new information most probably arise from dysfunctional hippocampal neurons and impaired synaptic plasticity and memory (Milnerwood et al., 2006; Puigdellívol et al., 2016; Smith‐Dijak et al., 2019). Disease-dependent misregulation of BDNF and its downstream signaling pathways in HD models and patients have been well-described (Smith‐Dijak et al., 2019) and BDNF is one of the master regulators of synaptic plasticity (Park and Poo, 2013). It has been shown that both loss of wt and gain of mutant HTT in HD decreases the levels of the neurotrophic factor BDNF (Zuccato et al., 2001, 2003; Gauthier et al., 2004) and increasing BDNF signaling rescues synaptic plasticity and memory in HD mice (Lynch et al., 2007; Anglada-Huguet et al., 2016). It has been recently reported that TCF4 regulates *Bdnf* expression (Tuvikene et al., 2021), and here we report downregulation of both TCF4 and *Bdnf* expression in the hippocampus and cerebral cortex of R6/1 mice. It is plausible that the downregulation of TCF4 could be one of the causal mechanisms underlying the downregulation of BDNF expression in the hippocampus and cerebral cortex of HD mouse models and patients in addition to the well-described NRSF-dependent mechanism (Zuccato et al., 2001, 2003, 2007; Conforti et al., 2013).

Spatial memory is believed to rely on adult hippocampal neurogenesis (Abrous and Wojtowicz, 2015) and impairment of spatial perception and spatial memory are reported in HD patients, correlating with their HD disease burden score (Harris et al., 2019). Additionally, R6/1 and R6/2 HD mice and the more slowly progressing YAC128 HD mice show reduced hippocampal adult neurogenesis in the SGZ of DG (Lazic et al., 2004; Gil et al., 2005; Simpson et al., 2011). Considering (1) the functions of TCF4 in neurogenesis (Schoof et al., 2020; Wang et al., 2020) and neural plasticity (Kennedy et al., 2016; Thaxton et al., 2018; Badowska et al., 2020); (2) high expression of TCF4 in hippocampal neuroepithelium and its persistence in all mature hippocampal neuron subpopulations and astrocytes and similar high expression in cortical structures (Jung et al., 2018; Kim et al., 2020); and (3) downregulation of TCF4 we show here in R6/1 mouse and HD patient hippocampus and cerebral cortex, allows to hypothesize that TCF4 might play a role in the impairment of cognitive functions in HD. In R6/1 mice, the motor and cognitive symptoms generally appear at 12–20 weeks of age (Naver et al., 2003; Bolivar et al., 2004; Giralt et al., 2013), and treatment with papaverin (inhibitor of PDE10a) or betulinic acid improve these symptoms (Giralt et al., 2013; Alcalá‐Vida et al., 2021). Importantly, both of these compounds affect PKA and cAMP levels, which are known to increase TCF4 transcriptional activity (Sepp et al., 2017). It is tempting to speculate that this effect could be, at least partially, be because of the increased TCF4-dependent gene expression.

To conclude, we have identified TCF4 as a dysregulated transcription factor in HD and provided evidence for the brain region-dependent regulation and functions of alternative TCF4 isoforms. Future work will elucidate the functional role of this dysregulation.

## Synthesis

Reviewing Editor: Eran Perlson, Tel Aviv University Decisions are customarily a result of the Reviewing Editor and the peer reviewers coming together and discussing their recommendations until a consensus is reached. When revisions are invited, a fact-based synthesis statement explaining their decision and outlining what is needed to prepare a revision will be listed below. The following reviewer(s) agreed to reveal their identity: Brady Maher.

The manuscript titled “Isoform-specific reduction of the bHLH transcription factor TCF4 levels in Huntington's disease” identified TCF4 as a transcription factor that is reduced in an isoform-specific manner in cell lines, animal models, and drug-induced models of Huntington’s as well as in postmortem hippocampal samples of human Huntington’s patients. Given the critical role of TCF4 in neurodevelopment, authors suggest that TCF4 might be one of the mediators of these alterations in different brain regions in presymptomatic Huntington’s. The manuscript is very well written and the data, in general, are straightforward and clean.

The work is a potentially a good fit for publications in eNeuro after addressing and clarifying some concerns:

1- The authors claimed that they identified TCF4 as a candidate that is altered in cell lines carrying the Huntington’s mutation among more than 200 transcription factors, however, no data was provided for this initial screen. Please provide data for the initial screen as a new figure and explain/justify why TCF4 was proceeded with if there were more than one hit in this screen.

2- Authors stated that they found a reduced “nuclear” signal for TCF4 in cell lines expressing Htt alleles with repeats (Fig 1A). However, the difference in TCF4 expression is not apparent in the printed image, and quantification is required.

3. Authors stated that Hdh7/109 and Hdh109/109 lysates showed about a 30% decrease in lower molecular weight isoforms of TCF4, while the grossly visible decrease in higher molecular weight isoforms (that is apparent from the representative blot) was not statistically significant. However, the significant decrease in lower molecular weight isoforms seems much larger than 30% and looks quite comparable with the decrease in higher molecular weight isoforms (Fig 2C). There are two concerns about this experiment. The first is that with semiquantitative methods, it would be wiser to have a larger sample size than what is traditionally accepted as the lowest minimum (n=3), especially when the decrease in higher molecular weight isoforms is quite grossly appreciable. This is quite important given that the paper claims that the reduction in TCF4 is “isoform-specific”. The second is that the absence of error bars for the control group that originates from normalization (Fig 2D). It is a better practice to normalize all data points (including those of the control group) to the mean of the control group to calculate the standard deviation and/or standard error of the mean for the control group as well. If authors are willing to add more data points per group, log transformation may not be needed to analyze the immunoblots as the change is unidirectional, as well as the use of parametric test can be justified if the sample size is increased without a need for log-transformation. Same concerns are also valid for all immunoblot quantification throughout the paper. This is not critical, however it seems like the authors missed some of the real changes (provided p values are just above 0.05 with quite robust % decrease) because of the concerns conveyed above. Addressing these concerns would definitely increase the credibility/impact of the manuscript. Adding st-eror to the controls will be good.

4. The authors showed isoform-specific changes in TCF4 transcript and protein levels in the cortex, striatum, and hippocampus of the mouse model, however only hippocampal levels were presented from postmortem brain tissues. Please provide an explanation why the other brain regions were skipped in the latter. If possible, we know it is not easy to get these samples, please provide data for the cortex and striatum as well.

5. Line 75 - missing parentheses
